# MART-10, a New Generation of Vitamin D Analog, Is More Potent than 1**α**,25-Dihydroxyvitamin D_3_ in Inhibiting Cell Proliferation and Inducing Apoptosis in ER+ MCF-7 Breast Cancer Cells

**DOI:** 10.1155/2012/310872

**Published:** 2012-12-11

**Authors:** Kun-Chun Chiang, Chun-Nan Yeh, Shin-Cheh Chen, Shih-Che Shen, Jun-Te Hsu, Ta-sen Yeh, Jong-Hwei S. Pang, Li-Jen Su, Masashi Takano, Atsushi Kittaka, Horng-Heng Juang, Tai C. Chen

**Affiliations:** ^1^General Surgery Department, Chang Gung Memorial Hospital, 222 Mai-Chin Road, Keelung 204, Taiwan; ^2^Graduate Institute of Clinical Medical Sciences, College of Medicine, Chang Gung University, Kwei-Shan, Taoyuan 333, Taiwan; ^3^General Surgery Department, Chang Gung Memorial Hospital, Kwei-Shan, Taoyuan 333, Taiwan; ^4^Institute of Systems Biology and Bioinformatics, National Central University, Jhongli City, Taoyuan 32001, Taiwan; ^5^Faculty of Pharmaceutical Sciences, Teikyo University, 2-11-1 Kaga, Itabashi, Tokyo 173-8605, Japan; ^6^Department of Anatomy, College of Medicine, Chang Gung University, 259 Wen-Hua 1st Road, Kwei-Shan, Taoyuan 333, Taiwan; ^7^Department of Medicine, Boston University School of Medicine, Boston, MA 02118, USA

## Abstract

Hormone antagonist therapy for estrogen receptor positive (ER+) breast cancer patients post radical surgery and radiation therapy has a poor prognosis and also causes bone loss. 1**α**,25-dihydroxyvitamin D_3_ [1**α**,25(OH)_2_D_3_] is a potent antitumor agent in pre-clinical studies, but caused hypercalcemia when its effective antitumor doses were used. Therefore, we investigated the effects of a less-calcemic 1**α**,25(OH)_2_D_3_ analog, 19-nor-2**α**-(3-hydroxypropyl)-1**α**,25-dihydroxyvitamin D_3 _(MART-10), on ER+MCF-7 cells. We demonstrate that MART-10 is 500- to 1000-fold more potent than 1**α**,25(OH)_2_D_3_ in inhibiting cell growth in a dose- and time-dependent manner. MART-10 is also much more potent in arresting MCF-7cell cycle progression at G_0_/G_1_ phase as compared to 1**α**,25(OH)_2_D_3_, possibly mediated by a greater induction of p21 and p27 expression. Moreover, MART-10 is more active than 1**α**,25(OH)_2_D_3_ in causing cell apoptosis, likely through a higher BAX/Bcl expression ratio and the subsequent cytochrome C release from mitochondria to cytosol. Based on our *in vitro* findings, MART-10 could be a promising vitamin D analog for the potential treatment of breast cancer, for example, ER+ patients, to decrease the tumor relapse rate and the side effect on bone caused by antihormone regimens. Thus, further *in vivo* animal study is warranted.

## 1. Introduction

Breast cancer ranks first globally among the most commonly diagnosed and cancer-related deaths in women [1]. Over 1.38 million new breast cancer cases and 458,400 breast cancer-related deaths have been reported worldwide in 2008. Estrogen receptor (ER), which is present in nearly 70% of all breast cancer patients, plays a crucial role in the progression of breast cancer [2]. Thus, ER antagonists, tamoxifen and raloxifene, have been widely used to treat breast cancer and have contributed to a better prognosis for ER positive (ER+) breast cancer. However, only a 50% reduction in tumor relapse has been achieved by ER antagonist therapy [3]. Furthermore, the antagonists have serious side effects on bone [4], which highlights the necessity of seeking alternative treatments for ER+ breast cancer. 

Vitamin D is well known as a modulator of calcium and bone metabolism. For the past three decades, abundant evidence has been accumulated to indicate that the active form of vitamin D, 1*α*,25-dihydroxyvitamin D_3_, 1*α*,25(OH)_2_D_3_, or calcitriol, possesses many actions not associated with calcium and bone metabolism [5]. They include antiproliferation, antiangiogenesis, proapoptosis, prodifferentiation, and immune regulation in a cell- and tissue- specific manner [5–9]. 

1*α*,25(OH)_2_D_3_ exerts its effects through binding to vitamin D receptor (VDR). The receptor is expressed in most human cancer cell lines and its growth can be inhibited by 1*α*,25(OH)_2_D_3_ [10–14]. However, the clinical application of 1*α*,25(OH)_2_D_3_ is hindered by its lethal hypercalcemic side-effect after its systemic administration at a concentration sufficient to inhibit tumor cell growth [15]. To overcome this drawback, thousands of vitamin D analogs have been synthesized aiming to minimize its calcemic side effect while maintaining or even potentiating the antitumor activities [16, 17].

For breast cancer, 1*α*,25(OH)_2_D_3_ and its analogs, including EB1089, ILX 23-7533, and 22-oxa-1*α*,25(OH)_2_D_3_, have been shown to be effective in suppressing breast cancer cell growth *in vitro* and *in vivo *either alone or in combination with other drugs [18]. However, no significant benefit on survival has been observed in clinical trials [19, 20].

MART-10 (19-nor-2*α*-(3-hydroxypropyl)-1*α*,25-dihydroxyvitamin D_3_) [21] has been shown to be more active in VDR transactivation [22]. Most importantly, MART-10 is far more potent in inhibiting liver and prostate cancer cell proliferation [11, 22, 23] and prostate cancer cell invasion [24], and it did not raise serum calcium *in vivo* in an animal model [24]. These findings suggest that MART-10 could be a good candidate for breast cancer treatment. We, therefore, study the antiproliferative and proapoptotic effects of MART-10 in ER+ MCF-7 breast cancer cells and the potential mechanisms involved. 

## 2. Materials and Methods

### 2.1. Vitamin D Compounds

1*α*,25(OH)_2_D_3_ was purchased from Sigma (St. Louis, MO, USA). MART-10 was synthesized as previously described [21].

### 2.2. Cell Culture

Human breast cancer cell lines, MCF-7 and MDA-MB-231, were obtained from Bioresource Collection and Research Center (BCRC, Taiwan). Both MCF-7 and MDA-MB-231 cells were grown in DMEM (Sigma) supplemented with 5% fetal bovine serum (FBS). Culture medium was changed 3 times per week.

### 2.3. Cell Proliferation Assay by Cell Number Counting

Cell counting was conducted using a hemocytometer as previously described [11]. Cells were treated every two days and counted on day 7.

### 2.4. Western Blot for Protein Expression

The procedures for protein extraction, blocking, and detection were described previously [11]. The primary antibodies used in this study were monoclonal antibodies against VDR (D-6, Santa Cruz Biotechnology, Santa Cruz, CA, USA), p21 (2946, Cell Signal, Beverly, MA, USA), p27 (3698, Cell Signal), cytochrome C (clone 7H8.2C12, BD Biosciences Pharmingen), Bax (554104, BD), and Bcl-2 (05-729, Millipore, Bedford, MA, USA). The secondary antibodies (1 : 5000) were anti-rabbit (111-035-003, Jackson Immunoresearch, West Grove, PA, USA) or anti-mouse secondary antibodies (Zymed 81-6520). The blots were detected using ECL reagents (WBKLS0500, Millipore, Billerica, MA, USA). Membranes were detected by VersaDoc Imaging System (Bio-Rad, Hercules, CA, USA) for analysis. 

### 2.5. Cell Cycle Analysis by Flow Cytometry

Flow cytometry for cell cycle analysis was performed using a FACSCalibur (BD Biosciences, San Jose, CA, USA) as described previously [11, 25]. Briefly, after exposure for two days to indicated concentrations of 1*α*,25(OH)_2_D_3_, the cells were collected and fixed in ice-cold 75% ethanol at 20°C overnight. The fixed cells were stained in propidium iodide (PI) buffer containing 100 mM sodium citrate, 0.1% Triton X-100, 0.2 mg/mL RNase, and 50 *μ*g/mL PI at 4°C for 1 h. Flow cytometry and cell cycle analysis were then performed using a FACSCalibur.

### 2.6. Apoptosis Analysis by Flow Cytometry

MCF-7 cell apoptosis was analyzed using a flow cytometer with Annexin V-FITC (fluorescein isothiocyanate) and propidium iodide (PI) staining kit (Strong Biotech Corporation, Taiwan) to distinguish early apoptotic from necrotic cells as previously described [11, 26]. Briefly, three days after the indicated concentrations of MART-10 or 1*α*,25(OH)_2_D_3_ treatment, MCF-7 cell apoptosis was analyzed using a flow cytometer with Annexin V-FITC (fluorescein isothiocyanate) and propidium iodide (PI) staining. Apoptosis Detection Kit (Strong Biotech Corporation, Taiwan) was applied in the present study. Briefly, cells from each sample were suspended in a mixture of 2 *μ*L Annexin V-FITC, 2 *μ*L propidium iodide (PI), and 100 *μ*L AnnexinV-FITC binding buffer and then incubated at room temperature for 15 min. According to the cell density, 0.4–0.8 mL binding buffer was added. The samples were analyzed using a flow cytometer FACS Calilbur (BD Biosciences). The cell population was separated into three groups, that is, live cells with a low level of fluorescence, apoptotic cells in the earlier period with green fluorescence (Annexin V positive), and necrotic and advanced stage apoptotic cells with both red and green fluorescence (Annexin V and PI positive).

### 2.7. Apoptosis Analysis by TUNEL Assay

TUNEL assay was used to measure DNA fragmentation [27]. Briefly, cells were plated on autoclaved glass coverslips in six-well culture plates and treated with MART-10 or 1*α*,25(OH)_2_D_3_ as indicated in the figure legends. Cellular DNA was stained with apoptosis detection kits (Millipore Billerica, MA, USA), and the assay was performed according to the recommendations from the manufacturer (Millipore Billerica). 

### 2.8. Statistical Analysis

The data from each group were compared by the student *t-*test. *P-*value < 0.05 was considered as a significant difference. Functions of Excel 2007 were used to calculate test statistics.

## 3. Results 

### 3.1. VDR Expression in MCF-7 Cells

**Figure 1:**
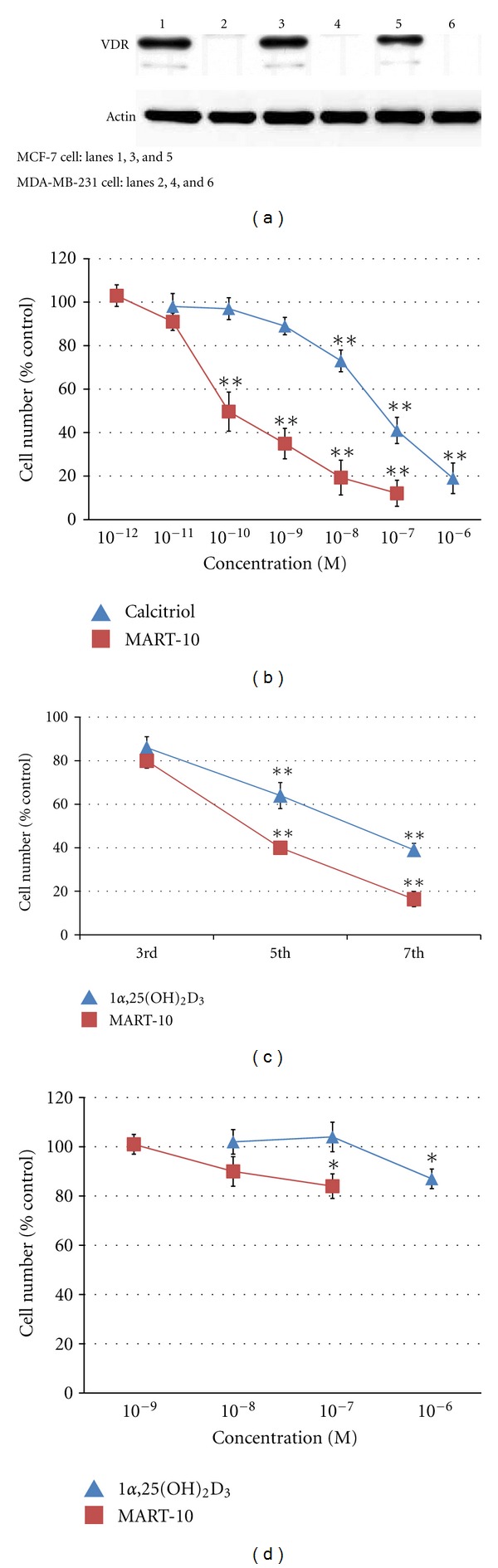
VDR expression in MCF-7 cells and MDA-MB-231 cells and the antiproliferative activity of 1*α*25(OH)_2_D_3 _and MART-10 in MCF-7 cells. (a) VDR expression in MCF-7 (lanes 1, 3, and 5) and MDA-MB-231 cells (lanes 2, 4, and 6) as determined by western blot method. Sixty *μ*g proteins were added in each lane. VDR was expressed much more prominently in MCF-7 than in MDA-MB-231 cells. (b) The dose-dependent inhibitory effects of 1*α*25(OH)_2_D_3_ and MART-10 on the growth of MCF-7 cells. Cells were plated at 5,000 cells per cm^2^ in 35 mm dishes. Two days after plating, cells were treated with 1*α*25(OH)_2_D_3_ or MART-10 for 1 week at the indicated concentrations as described in the Materials and Methods. Cell numbers were obtained using a hemocytometer. Results are presented as the percentage of control. Each value is a mean ± SD of three to five determinations. *P < 0.05, **P < 0.001 versus control. (c) The time-dependent inhibitory effects of 1*α*25(OH)_2_D_3_ and MART-10 on the growth of MCF-7 cells. Cells were grown and treated with 1*α*25(OH)_2_D_3_ or MART-10 at the indicated concentrations two days after plating and counted on days 3, 5, and 7, respectively. Cell numbers were obtained using a hemocytometer. Results are presented as the percentage of control. Each value is a mean ± SD of three to five determinations. *P < 0.05, **P < 0.001 versus control. (d) The dose-response effects of 1*α*,25(OH)_2_D_3_ and MART-10 treatment on the growth of MDA-MB-231 cells. Cells plated at 5,000 cells per cm^2^ in 35 mm dishes were grown and treated with 1*α*,25(OH)_2_D_3_ or MART-10 at indicated concentrations for 1 week two days after plating as described in the Materials and Methods. Cell numbers were obtained using a hemocytometer. Results are presented as the percentage of control. Each value is a mean ± SD of three to five determinations. *P < 0.05, **P < 0.001 versus control.

Since the genomic actions of 1*α*,25(OH)_2_D_3_ are mediated through VDR, we first analyzed the expression of VDR in MCF-7 cells. The expression in MDA-MB-231 cells served as a negative control [28]. As demonstrated in Figure 1(a), VDR was highly expressed in MCF-7 cells (lanes 1, 3, and 5), whereas very little or no expression (lanes 2, 4, and 6) was found in MDA-MB-231 cells as previously reported [28]. 

### 3.2. Antiproliferative Effect of MART-10 and 1*α*,25(OH)_2_D_3_ on MCF-7 Cells

To compare the antiproliferative activity of MART-10 and 1*α*,25(OH)_2_D_3_ in MCF-7 cells, the cells were treated with either MART-10 or 1*α*,25(OH)_2_D_3_, and the cell numbers were counted on 7th day as previously described [11]. As shown in Figure 1(b), either 1*α*,25(OH)_2_D_3_ or MART-10 caused a dose-dependent inhibition of cell growth. However, MART-10 caused a 50 ± 9% inhibition at 10^−10^ M, whereas, no inhibition was observed with 10^−10^ M of 1*α*,25(OH)_2_D_3_. Only when 10^−7^ M 1*α*,25(OH)_2_D_3_ was used, a 58 ± 6% inhibition was obtained. Thus, it is concluded that MART-10 is about 500- to 1000-fold as potent as 1*α*,25(OH)_2_D_3_ to repress MCF-7 cell growth. 

Figure 1(c) shows a time course inhibition of MCF-7 cell growth by 1*α*,25(OH)_2_D_3_ and MART-10 at 10^−7^ M. 1*α*,25(OH)_2_D_3_ inhibited MCF-7 cell growth by 14 ± 5, 46 ± 6 and 61 ± 3% on the 3rd, 5th, and 7th day, whereas a 20 ± 3, 60 ± 3, or 84 ± 4% growth inhibition by MART-10 was observed at the same time points. A greater inhibition by MART-10 was observed at each time point.

Figure 1(d) demonstrates that MDA-MB-231 cells were not as responsive as MCF-7 cells to 1*α*,25(OH)_2_D_3_ and MART-10 treatments. Only a 13 ± 6% and a 16 ± 5% inhibition were observed in the presence of 10^−6^ M 1*α*,25(OH)_2_D_3_ and 10^−7^ M MART-10, respectively. The results are in agreement with the VDR expression data obtained by western blot analysis showing much less expression of VDR in MDA-MB-231 cells than in MCF-7 cells (Figure 1(a)).

### 3.3. Induction of Cell Cycle Arrest at G_0_/G_1_ Phase and the Cyclin Dependent Kinase (CDK) Inhibitors, p21 and p27, by MART-10 and 1*α*,25(OH)_2_D_3_ in MCF-7 Cells

**Figure 2:**
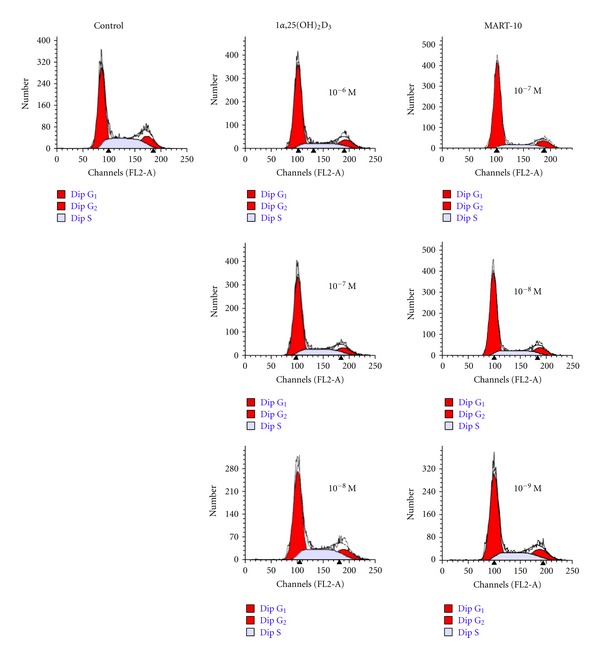
Flow cytometry analysis of cell cycle distribution for MCF-7 cells treated by 1*α*25(OH)_2_D_3_ and MART-10. Effects of 1*α*25(OH)_2_D_3_ and MART-10 on the relative distribution of MCF-7 cells at G_1_/G_0_, S and G_2_/M phase. MCF-7 cells were treated with 1*α*25(OH)_2_D_3_ from 10^−8^ M to 10^−6^ and MART-10 from 10^−9^ M to 10^−7^ M for two days before cell cycle analysis was performed with a flow cytometer. A representative DNA histogram for control, 1*α*25(OH)_2_D_3_-, or MART-10-treated MCF-7 cells was shown. The total DNA content of cells (*x*-axis) was obtained by staining with propidium iodide. Cells were analyzed by flow cytometry. The percentage of cells in each cell cycle phase was determined with the program ModFit. The first large peak represents population of cells (*y*-axis) in G_0_/G_1_ phase, the second small peak shows population of cells in G_2_/M phase, and the gray area between both peaks represents cells in S phase.

Since MART-10 and 1*α*,25(OH)_2_D_3_ showed a significant inhibition in the growth of MCF-7 cells, we next conducted cell cycle analysis by flow cytometry to further understand the mechanisms mediating the inhibition. When MCF-7 cells were treated with 10^−8^, 10^−7^, and 10^−6^ M 1*α*,25(OH)_2_D_3_ for two days_, _the fraction of cells arrested at G_0_/G_1_ phase increased by 5.81%, 13.34%, and 13.78%, respectively, whereas we observed an increase in cell arrest at G_0_/G_1_ by 10.45%, 15.36%, and 19.93% in the presence of 10^−9^, 10^−8^, and 10^−7^ M of MART-10, respectively, as compared to the controls (Figure 2 and Table 1). It is clear that although either 1*α*,25(OH)_2_D_3_ or MART-10 can significantly arrest MCF-7 cell cycle progression at G_0_/G_1_, MART-10 is much more potent than 1*α*,25(OH)_2_D_3_ in this respect. 

**Figure 3:**
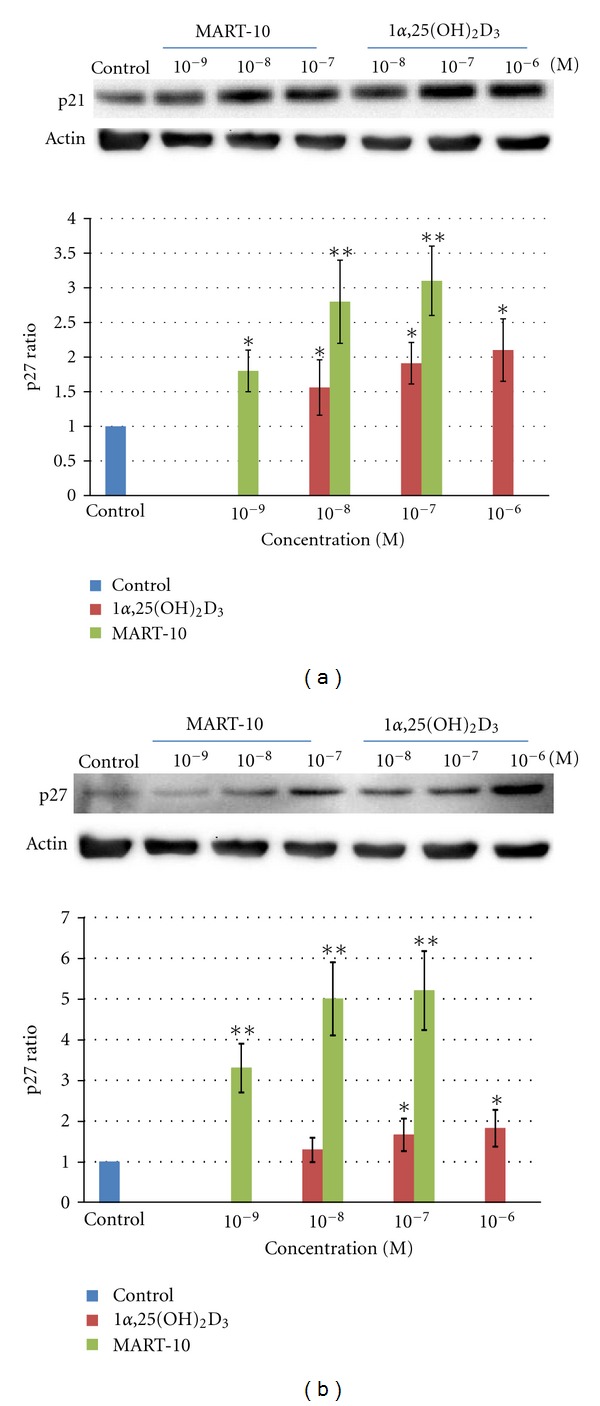
Western blot analysis for the expressions of p21 and p27 after treating MCF-7 cells with 1*α*25(OH)_2_D_3_ and MART-10. (a) A western blot (30 *μ*g protein was loaded for each individual lane) depicting a typical dose-dependent upregulation of p21 protein expression in response to the treatment with 1*α*25(OH)_2_D_3_ or MART-10 for 2 days (upper panel). Actin was used as the loading control. The lower panel shows the average radio of the dose-dependent p21 expression relative to actin expression from three independent experiments. Each value is a mean ± SD of three independent determinations. (b) A western blot (30 *μ*g protein was loaded for each individual lane) depicting a typical dose-dependent up-regulation of p27 protein expression in response to the treatment with 1*α*25(OH)_2_D_3_ or MART-10 for 2 days (upper panel). Actin was used as the loading control. The lower panel depicts the average radio of the dose-dependent p27 expression relative to actin expression from three independent experiments. Each value is a mean ± SD of three independent determinations. *P < 0.05, **P < 0.001 versus control.

Since p21 and p27 have been implicated in the G_0_/G_1_ arrest by 1*α*,25(OH)_2_D_3_, we next examined the expression of p21 and p27 in the presence of 1*α*,25(OH)_2_D_3_ or MART-10 by western blot analysis.  Figure 3(a) demonstrates that p21 expression increased 1.56 ± 0.4, 1.91 ± 0.3, and 2.1 ± 0.45 time over the control group, after treating with 10^−9^, 10^−8^, and 10^−7^ M of 1*α*,25(OH)_2_D_3_ for two days, respectively, whereas 1.8 ± 0.3, 2.8 ± 0.6, and 3.1 ± 0.5 fold expressions were induced by MART-10 at 10^−9^, 10^−8^, and 10^−7^ M, respectively. As for p27 expression, 1*α*,25(OH)_2_D_3_ induced 1.29 ± 0.3, 1.66 ± 0.4, and 1.82 ± 0.45 time over the controls upon treatment with 10^−8^, 10^−7^, and 10^−6^ M of 1*α*,25(OH)_2_D_3_ for two days, respectively. MART-10 at 10^−9^, 10^−8^, and 10^−7^ M upregulated p27 expression 3.3 ± 0.6, 5 ± 0.9, and 5.3 ± 0.97 fold over the controls (Figure 3(b)). Taken together, we conclude that 1*α*,25(OH)_2_D_3_ and MART-10 are both able to upregulate p21 and p27 expression in a dose-dependent manner, and MART-10 is much more potent than 1*α*,25(OH)_2_D_3_.

### 3.4. Effects of 1*α*,25(OH)_2_D_3_ and MART-10 on MCF-7 Cell Apoptosis and Apoptotic Protein Expression

**Figure 4:**
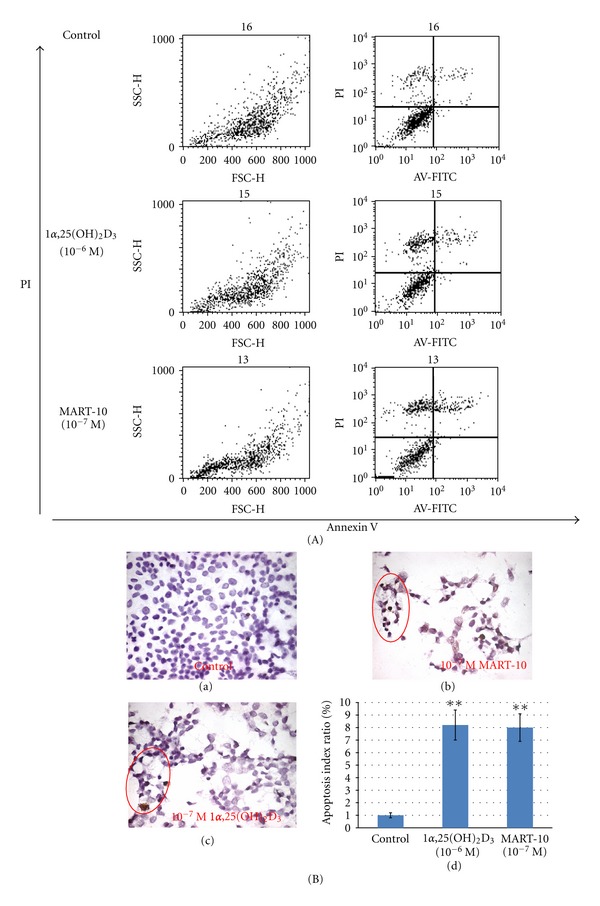
Effects of 1*α*25(OH)_2_D_3_ and MART-10 on MCF-7 cell apoptosis analyzed by flow cytometry with Annexin V-FITC, PI staining, and TUNEL assay. (A) Annexin V-FITC in conjunction with PI staining was used to distinguish early apoptotic (Annexin V-FITC positive, PI negative; bottom right quadrant of each panel) from late apoptotic or necrotic cells (Annexin V-FITC positive, PI positive; top right quadrant of each panel). Fluorescence intensity for Annexin V-FITC is plotted on the *x*-axis, and PI is plotted on the *y*-axis. (B) The apoptotic effects induced by MCF-7 cells were analyzed by TUNEL assay to measure the extent of DNA fragmentation visualized by fluorescence microscopy: (a) control; (b) cells treated with 10^−7^ M MART-10; (c) cells treated with 10^−7^ M 1*α*25(OH)_2_D_3_. The cells showing positive DNA fragmentation were circled; (d) relative apoptotic index. Each value represents the average of three determinations. *P < 0.05, **P < 0.001 versus control.

To compare the apoptotic response induced by 1*α*,25(OH)_2_D_3_ and MART-10 in MCF-7 cells, flow cytometry analysis coupled with staining cells with Annexin V (Annexin V-FITC) and PI was utilized [29] (Figure 4(A)). The quantitative numerical distribution of apoptotic cells from this analysis is presented in Table 2. 1*α*,25(OH)_2_D_3_ at 10^−6^ M induced MCF-7 cell apoptosis by increasing the late apoptotic cell population from 7.19% to 10.04%, while MART-10 at 10^−7^ M was able to increase the late apoptosis cell population from 7.19% to 13.66%. The results are in agreement with those obtained by TUNEL assay (Figure 4(B), panels a, b, c, and d). The figure shows that 8.2% and 8% apoptotic cells were generated when MCF-7 cells were treated with 10^−6^ M 1*α*,25(OH)_2_D_3_ and 10^−7^ M MART-10, respectively. Our results, therefore, indicate that MART-10 is about 10-fold more potent than 1*α*,25(OH)_2_D_3_ in the apoptotic induction of MCF-7 cells.

**Figure 5:**
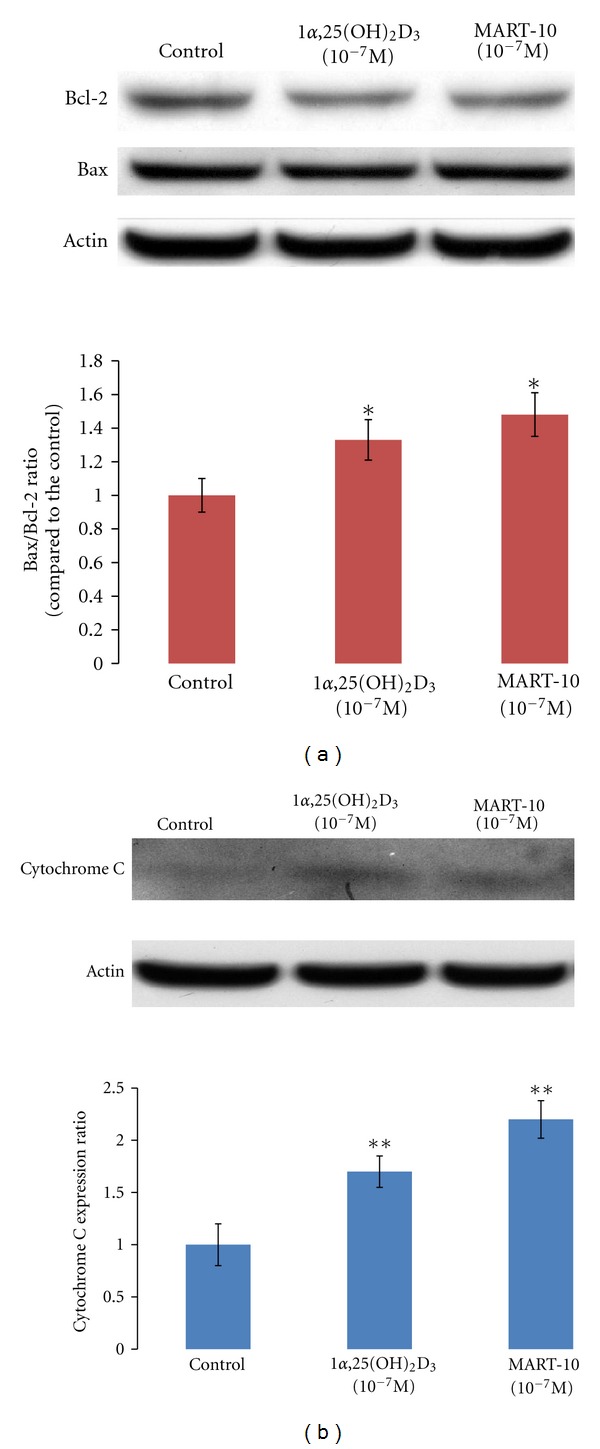
Effects of 1*α*25(OH)_2_D_3_ and MART-10 on the protein expression of Bcl-2, Bax, and cytochrome C in MCF-7 cells. (a) Western blot analysis of Bcl-2 and Bax expression in the untreated control, and cells treated with either 10^−7^ M of 1*α*25(OH)_2_D_3_ or MART-10 for 5 days (upper panel). Thirty *μ*g protein was loaded in each lane. The lower panel depicts the calculated BAX/Bcl-2 ratio obtained from scanning the bands shown in the upper panel based on the control Bax/Bcl-2 ratio set as 1. (b) Western blot analysis of cytochrome C expression in cytosol after treatment with ethanol vehicle, 1*α*25(OH)_2_D_3_, or MART-10 (upper panel) and the expression ratio over the control (lower panel). Each value represents the average of three determinations. *P < 0.05, **P < 0.001 versus control.

Bax protein is a well-known proapoptotic protein, whereas Bcl-2 is a protein with antiapoptotic activity. Therefore, the higher Bax/Bcl-2 ratio has been used as an indicator for the expression and the subsequent release of cytochrome C into cytosol to trigger apoptosis. As shown in Figure 5(a), 10^−7^ M MART-10 and 1*α*,25(OH)_2_D_3_ increased the Bax/Bcl-2 ratio to 1.48 and 1.33 as compared to the controls, which is in agreement with a greater upregulation of cytochrome C expression over controls by MART-10 (2.35-fold) than by 1*α*,25(OH)_2_D_3_ (1.64-fold) (Figure 5(b)). 

## 4. Discussion 

The focus of this study was to investigate the antiproliferative and proapoptotic activities of MART-10 in the ER+ MCF-7 breast cancer cells which express high level of VDR (Figure 1(a)). MART-10 is a new generation of 1*α*,25(OH)_2_D_3_ analogs with a skeleton of “2*α*-(3-hydroxy)propyl group” and “19-nor” integrated into one molecule. Therefore, MART-10 possesses the combined characteristics of the noncalcemic nature of the 19-nor vitamin D compounds [30] as exemplified by the FDA-approved drug Zemplar or 19-nor-1*α*, 25(OH)_2_D_2_ for the treatment of the secondary hyperparathyroidism, and the enhanced VDR binding property of 2*α*-(3-hydroxy)propyl compound [31, 32]. Similar to Zemplar, MART-10 did not raise serum calcium in an *in vivo* animal model [23] and was more potent than 1*α*,25(OH)_2_D_3_ in inducing VDR transactivation [22].

The effects of vitamin D are mainly mediated through the VDR-dependent genomic actions. Our results confirm the high level of VDR expression in MCF-7 cells and accordingly highly sensitive growth inhibitory responses to 1*α*,25(OH)_2_D_3_ and MART-10 in a dose- and time-dependent manner (Figures 1(b) and 1(c)). The low or no expression of VDR in MDA-MB-231 cells (Figure 1(a)) is in agreement with the low antiproliferative activity caused by 1*α*,25(OH)_2_D_3_ and MART-10 (Figure 1(d)) in these VDR-null cells. Thus, the results clearly suggest that VDR plays a crucial role in the response of MCF-7 breast cancer cells to 1*α*,25(OH)_2_D_3_. _._Along this line, Lopes et al. recently reported that VDR expression was high in benign breast lesions and diminished gradually in invasive breast cancer as the tumor progressed [33]. VDR expression has also been shown to be inversely related to breast cancer incidence [34]. Collectively, the findings suggest that dysregulation of VDR expression may contribute to the incidence and progression of breast cancer. 

In addition, our data, showing a greater cell growth inhibition induced by MART-10 than by 1*α*25(OH)_2_D_3_ on day 5 and day 7 (Figure 1(c)), suggest that the effective dose of MART-10 may be higher than that of 1*α*,25(OH)_2_D_3_, possibly because MART-10 is more bioavailable than 1*α*,25(OH)_2_D_3_ due to the nature that MART-10 is more resistant to CYP24A1 degradation [22, 23].

Our results show that although both 1*α*,25(OH)_2_D_3_ and MART-10 are active in inhibiting the proliferation (Figures 1(b) and 1(c)), inducing the cell cycle arrest at G_0_/G_1_ phase (Figure 2 and Table 1) and promoting the apoptosis of MCF-7 cells (Figure 4), MART-10 is far more potent than 1*α*,25(OH)_2_D_3_. The greater antiproliferative activity with MART-10 over 1*α*,25(OH)_2_D_3_ may be explained at least in part by its greater stimulatory effects on the expression of two tumor suppressor genes, p21 and p27, which act as CDK inhibitors to inhibit the progression of cells into the S phase of the cell cycle (Figure 3). This finding is consistent with several previous reports that showed that p21 and p27 were the genes targeted by 1*α*25(OH)_2_D_3_ and, therefore, leading to the arrest of cell growth [11, 35, 36].

As demonstrated in Figure 4 and Table 2, MART-10 is also more active than 1*α*,25(OH)_2_D_3_ in inducing apoptosis. Bax, a proapoptotic protein, works toward the initiation of apoptosis through promoting the release of cytochrome C from mitochondria into cytosol. Whereas, Bcl-2, an antiapoptotic protein, functions as a protector to stabilize the mitochondrial membrane from releasing cytochrome C [37]. Studying MCF-7 breast cancer cells, James et al. [38] and Simboki-Campbell et al. [39] reported that 1*α*,25(OH)_2_D_3_ induced apoptosis by downregulating Bcl-2 protein expression, increased TRPM-2 (clusterin) mRNA expression, and increased DNA fragmentation after 1*α*,25(OH)_2_D_3_ treatment. In our studies with MCF-7 cells, both 1*α*,25(OH)_2_D_3_ and MART-10 increased the ratio of Bax/Bcl-2 and the subsequent release of cytochrome C (Figures 5(a) and 5(b)). However, MART-10 is more potent than 1*α*,25(OH)_2_D_3_.

The release of cytochrome C from mitochondria to cytoplasm is a trigger of apoptosis pathway, leading to the activation of intrinsic initiator caspase 9, which in turn activates executioner caspase 3 and caspase 7 [40]. To investigate whether caspases were involved in the vitamin D-induced apoptosis in MCF-7 cells, we performed western blotting to detect the expression of the active form of caspases 3, 7, 8, and 9 in the presence of 10^−7^ M of 1*α*,25(OH)_2_D_3_ or MART-10 for 5 days. We found that none of them was detected either with or without 1*α*,25(OH)_2_D_3_ or MART-10 treatment (unpublished data). Our results are in agreement with the previously published observations by Narvaez and Welsh [41] and Jänicke et al. [42]. Collectively, we conclude that MART-10 and 1*α*,25(OH)_2_D_3_-mediated apoptosis in MCF-7 cells may be cytochrome C-related but caspases-independent, and MART-10 is more potent than 1*α*,25(OH)_2_D_3_ in inducing apoptosis in MCF-7 cells.

## 5. Conclusion

For premenopausal women with ER+ breast cancer, the choice for antihormone treatment is tamoxifen or raloxifene which binds to ER, whereas aromatase inhibitors are the major therapeutic antihormone agents for the postmenopausal women with ER+ breast cancer. The drawback of tamoxifen or raloxifene and aromatase inhibitors is that they globally attenuate estrogen receptor transactivation or estrogen synthesis. It may be undesirable for some tissues where estrogen is essential to maintain normal functions, such as bone which needs estrogen to stimulate bone formation. On the contrary, 1*α*,25(OH)_2_D_3_ can selectively down-regulate aromatase and ER-*α* expression in breast cancer cells [43, 44]. Along this line, we have performed preliminary studies indicating that MART-10 is far more potent than 1*α*,25(OH)_2_D_3_ in inhibiting ER-*α* expression in MCF-7 cells (unpublished observation). In conclusion, we show that MART-10 is much more potent than 1*α*,25(OH)_2_D_3_ in inhibiting cell growth through arresting cell cycle progression at G_1_ phase and inducing apoptosis. In addition, the more bioavailable character of MART-10 as compared to 1*α*,25(OH)_2_D_3_ in MCF-7 cells and its noncalcemic nature in an animal model suggest that MART-10 has potential as a superior chemotherapeutic agent to replace or to be in combination with traditional antihormone therapy for the treatment of breast cancer, such as the ER+ breast cancer patients, to decrease the tumor recurrence and eliminate the side effect on bone caused by the antihormone treatments. 

## Figures and Tables

**Table 1:** The distribution of different phases of MCF-7 cell cycle under the influence of 1*α*,25(OH)_2_D_3_ or MART-10.

	G_1_	S	G_2_/M
Control	50.36%	33.51%	16.13%
1,25D*, 10^−8^ M	56.17%	30.06%	13.77%
1,25D, 10^−7^ M	63.70%	23.85%	12.36%
1,25D, 10^−6^ M	64.14%	21.96%	13.90%
M-10^#^, 10^−9^ M	60.81%	23.65%	15.54%
M-10, 10^−8^ M	65.72%	21.32%	12.96%
M-10, 10^−7^ M	70.29%	12.98%	16.73%

*1,25D: 1*α*,25(OH)_2_D_3_.

^#^M-10: MART-10.

**Table 2:** The distribution of different phases of MCF-7 cell cycle after 1*α*,25(OH)_2_D_3_ or MART-10 treatment determined by flow cytometry with Annexin V-FITC and PI staining.

	PI negative, Annexin V negative	PI negative, Annexin V positive	PI positive, Annexin V positive
Control	76.22%	1.59%	7.19%
1*α*,25(OH)_2_D_3_ (10^−6^ M)	65.79%	1.19%	10.04%
MART-10 (10^−7^ M)	57.87%	1.76%	13.66%

## Abbreviations

ER: Estrogen receptor
1*α*,25(OH)_3_D: 1*α*,25-Dihydroxyvitamin D
MART-10: 19-nor-2*α*-(3-Hydroxypropyl)-1*α*,25(OH)_2_D_3_
VDR: Vitamin D receptor
VDRE: Vitamin D response element
RXR: Retinoid X receptor
PI: Propidium iodide
E2F-1: E2F transcription factor 1
FBS: Fetal bovine serum
FITC: Fluorescein isothiocyanate.
